# Like parents, like children… this is not always the case! A longitudinal study on the family transmission of intergroup contact

**DOI:** 10.1111/jora.13029

**Published:** 2024-10-15

**Authors:** Maria Pagano, Ioana Zagrean, Daniela Barni, Elisabetta Crocetti

**Affiliations:** ^1^ Department of Psychology Alma Mater Studiorum University of Bologna Bologna Italy; ^2^ Department of Human Sciences LUMSA University of Rome Rome Italy; ^3^ Department of Human and Social Sciences University of Bergamo Bergamo Italy

**Keywords:** developmental ecological contexts, family transmission, intergroup contact, longitudinal

## Abstract

During adolescence, opportunities for interethnic interactions can shape future attitudes toward diversity. However, it is unclear how family can influence adolescents' quality of contact in different life contexts. This study aims to fill this gap. A sample of 702 Italian adolescents (*M*_age_ = 15.61, SD_age_ = 1.11, 48.58% girls) and their parents (615 mothers, *M*_age_ = 48.45, SD_age_ = 4.34; 487 fathers, *M*_age_ = 51.22, SD_age_ = 4.92) completed questionnaires at two time points. Cross‐lagged models indicated that adolescents' intergroup contact at T1 was associated with mothers' contact over time, mainly in structured (i.e., school and work) contexts. No significant associations were found regarding fathers' intergroup contact and unstructured contexts. These results shed new light on the process of family transmission during adolescence, particularly regarding intergroup dynamics.

## INTRODUCTION

Multiculturalism is a defining characteristic of contemporary societies (UNHCR, 2022). In the developmental period of adolescence, young people have more opportunities to interact with peers and adults with different cultural backgrounds, and these experiences can shape their future attitudes toward diversity (e.g., Crocetti et al., 2021; Wölfer et al., 2016). While positive intergroup interactions foster social relationships and promote inclusion, negative interactions will likely hamper them (Tropp et al., 2022). The question is whether the roots of adolescents' quality of intergroup interactions can be traced to their family of origin or whether, on the contrary, it is adolescents who mainly influence their parents. Both adolescents and their parents come into contact with people of different ethnicities in everyday life contexts (e.g., school, leisure time, and work). For instance, adolescents from the majority group can meet and interact with their peers with a migrant background in the classroom or when they practice sports. Similarly, their parents can have intergroup interactions in their work environments and during activities practiced in their leisure time. Building on these premises, this study aims to understand how the quality of intergroup contact is transmitted within the family and how this transmission occurs in different life contexts.

### Intergroup contact as a way to foster social inclusion

Intergroup contact refers to interactions with people of an ethnic group other than one's own. Allport's (1954) seminal hypothesis suggests that intergroup contact is one of the keys to making societies more inclusive by fostering positive attitudes toward the outgroup and reducing prejudice. This happens when certain conditions are met: the two groups have the same social status, they cooperate to achieve a common goal, the contact is intimate, and authorities support it. Recent theoretical developments have emphasized that these conditions are facilitative for intergroup contact, albeit not all necessary (for a meta‐analysis, see Pettigrew & Tropp, 2006).

Furthermore, theoretical advances in intergroup contact research have emphasized the importance of considering not only the frequency (i.e., quantity) of intergroup contact but also the necessity to differentiate the *quality* of interactions (Hayward et al., 2017). Intergroup contact can have either positive (e.g., warm and respectful interactions) or negative (e.g., unfriendly and intimidating interactions) valence. Some demographic characteristics (i.e., age and sex) may influence the quality of these interactions. First, from a developmental point of view, in early adolescence, intergroup contact is more meaningful and positive, while in middle and late adolescence, it tends to be less stable and more negative (Wölfer et al., 2016). Second, females usually reported more positive and less negative contact (Bagci & Gungor, 2019; Karataş, Eckstein, et al., 2023b). Importantly, positive intergroup contact, which is generally more frequent than negative one (e.g., Hayward et al., 2017), can reduce negative attitudes toward the outgroup (e.g., Barni et al., 2020; Lutterbach & Beelman, 2023), whereas negative contact interactions are associated with higher levels of prejudice (e.g., Kotzur & Wagner, 2021; Schäfer et al., 2021). Therefore, it is of utmost importance to study how to foster frequent positive intergroup contact.

### Intergroup contact within the family context: The intergenerational transmission process

Since parents are one of the main agents of socialization, they can make their children more respectful of diversity through their example (Bagci & Gungor, 2019). Reciprocal influences happening within families can be understood through the lens of intergenerational transmission processes. *Intergenerational transmission* is a dynamic process leading to cultural continuity between generations across complex interactions among multiple aspects such as values, attitudes, norms, and behaviors (Meeus, 2016; Schönpflug & Bilz, 2009).

The family represents the first micro‐system in which child development occurs (Bronfenbrenner & Morris, 2006). It provides a privileged environment for learning values, attitudes, and behaviors (e.g., Knafo & Assor, 2007) and represents the primary socialization context. Consistent studies have shown that parents can act as role models for their adolescent children (Bandura, 1977), promoting parent–adolescent congruence in values, ideology, and behaviors (e.g., Goodman & Dyer, 2020; Perales et al., 2021). Fathers and mothers can exert different influences on their adolescent children. Indeed, adolescents are much more exposed to the example of mothers because they generally spend more time with them. At the same time, fathers can be more authoritarian and, consequently, children tend to conform more easily to their attitudes (Perales et al., 2021). Furthermore, regarding the parent–child transmission of issues related to ethnic and cultural diversity, Allport (1954) already supposed a direct transfer from parental words, emotions, and ideas to children's attitudes.

If, undoubtedly, parents have a profound impact on their children, family transmission processes are not unidirectional (from parents to children), but more likely they are reciprocal (between parents and children) or might even be reversed (from children to parents) (e.g., Barni et al., 2013; Kuczynski & Parkin, 2007). Schönpflug and Bilz (2009), in their Filter Model, pointed out that family transmission is a negotiation process regulated by the interactive contribution of parents and children. In this same line, transactional models of development (Sameroff, 2009) suggest that children can influence their parents, especially starting in adolescence, when the parent–child relationship becomes progressively more symmetrical (De Goede et al., 2009) and adolescents' persuasive strategies, cognitive skills, and critical thinking become more sophisticated.

Children can influence parents' values through various mechanisms (for a review, see Knafo & Galansky, 2008). For instance, this includes the active influence of children in encouraging changes in their parents' values, reciprocal influences between parents and children, or even counter‐influences where parental values shift in a direction opposite to their children's values. All the emerging literature on reverse socialization, mainly focused on environmental and technology literacy, is consistent in showing the active role of children in the transmission processes and their ability to unilaterally (through direct requests, explicit persuasive attempts, etc.) or bilaterally (through reasoning, negotiation, etc.) influence their parents (Singh et al., 2020).

Parent–child interaction and intra‐household information sharing seem to be the most important channels through which children's knowledge, attitudes, and behavior positively affect those of adults (e.g., Liu et al., 2022). Adolescents' agency in their interactions with parents is particularly pronounced in the case of ‘hot’ social issues, such as immigration, which are likely to give rise to more frequent and heated debates in families (Miklikowska, 2016). In this regard, Miklikowska (2016) showed that prejudice and tolerance toward immigrants mainly develop as a result of reciprocal influences between parents and adolescents.

Although some longitudinal evidence of children's influences and bidirectional effects has been documented, empirical results are still limited and inconclusive. Studies investigating the transmission of ideologies concerning people with migrant backgrounds (for a review, see Degner & Dalege, 2013) and ethnic prejudice (for a review, see Zagrean et al., 2022) are still predominantly cross‐sectional. Thus, on the one hand, only a few studies have employed a longitudinal research design to investigate the association between intergroup attitudes of adolescents and their parents (e.g., Hello et al., 2004; Miklikowska, 2016, 2017). On the other hand, these studies typically neglect the potential influence of children in support of a greater focus on the parent‐to‐children process (e.g., Gniewosz & Noack, 2015; Jugert et al., 2016). Moreover, to the best of our knowledge, no studies directly tackled whether intergroup contact is transmitted within the family environment and how this process operates.

### Intergroup contact in contexts

Adolescents and their parents can have diverse intergroup contact interactions in their life contexts. Distinguishing different but interdependent ecological contexts is essential (Bronfenbrenner, 1979; Sameroff, 2009) because adolescents' development occurs through mutual influences between oneself and the different settings in which they are embedded (e.g., family, school, and leisure time) and the primary agents of socialization within these contexts (e.g., parents, teachers, and peers). In addition, these contexts are differentiated by features that make them more or less structured. On the one hand, *structured contexts*, such as school for adolescents or work for parents, are contexts in which there are limited possibilities to choose the people with whom one enters into a relationship. On the other hand, leisure contexts (e.g., sports and groups of friends) are considered *unstructured contexts* in which choosing people with whom one comes into contact is simpler. In these different contexts, adolescents (Karataş, Eckstein, et al., 2023a) and adults (Schmid et al., 2017) can have multiple intergroup contact interactions, but how they are potentially interrelated is still unknown.

Furthermore, these micro‐contexts are embedded in the broader cultural macro‐context that can shape how the transmission process unfolds. Specifically, the current study was conducted in Italy, a nation that rapidly changed from a country of emigration into one of immigration in the late 1980s (Ceccon et al., 2023). The ethnic composition of the immigrant population within the country comprises different nationalities, with Romania (23%), Albania (8%), Morocco (8%), China (6%), and Ukraine (4%) being the primary countries of migration (ISTAT, 2023). Consequently, immigrant communities in Italy differ significantly from well‐established ethnic minorities, such as marginalized groups in the United States (e.g., African Americans). In this sense, the term ‘ethnic minorities’ refers to individuals who are either born abroad (e.g., first‐generation migrants or refugees) or have at least one parent born abroad (i.e., second‐generation migrants). In addition, the Italian school system distinguishes itself from other European countries through its universalist and inclusive approach, prioritizing integrating migrant children into regular classes and providing Italian language classes within school hours (European Commission/EACEA/Eurydice, 2019). Among students with migrant backgrounds enrolled in Italian schools, 65.3% of them are concentrated in the Northern part of Italy, specifically in the Emilia‐Romagna region where the present study took place (Ministero della Pubblica Istruzione, 2022). Interestingly, this pattern can be observed within secondary high schools, where 13.5% of students belong to ethnic minority groups, a significant increase compared to the national average of 8%. Consequently, the Emilia‐Romagna region is the most ethnically and culturally diverse in Italy. This increasing diversity improves opportunities for intergroup contact (Allport, 1954), especially in vocational schools where there are more students with migrant backgrounds than in academic‐oriented schools (Karataş, Eckstein, et al., 2023b).

## THE PRESENT STUDY

While extensive research has highlighted the unidirectionality of the transmission process (from parents to adolescents), less attention has been paid to the bidirectionality of the transmission (between parents and adolescents) regarding intergroup relationships, such as intergroup contact. On the one hand, mothers and fathers, via cultural transmission, might influence the intergroup contact quality of their adolescent child. On the other hand, adolescents from the majority group, being placed in multicultural contexts, can influence intergroup interactions with their parents. Thus, in line with the literature reviewed above, the current study aimed to investigate how the quality of intergroup contact is transmitted within the family context by examining the longitudinal reciprocal associations between adolescents' and their parents' positive and negative intergroup contact in different life contexts. Specifically, it is expected that intergroup interactions mothers and fathers have at work (structured setting) and during leisure time (unstructured setting) were bidirectionally associated with intergroup contact at school and during their adolescent children's leisure contexts over time. These associations were tackled by controlling for adolescents' age, sex, and school track because these factors were found to be related to adolescents' intergroup contact (e.g., Bagci & Gungor, 2019; Karataş, Eckstein, et al., 2023a, 2023b; Karataş, Rubini, et al., 2023; Wölfer et al., 2016).

## METHOD

### Participants

Participants for this study were drawn from the ongoing longitudinal project ‘IDENTITIES: Managing Identities in Diverse Societies: A Developmental Intergroup Perspective with Adolescents’. For the present study, 702 Italian adolescents (*M*_age_ = 15.61, SD_age_ = 1.11, 48.58% girls) and their parents (615 mothers, *M*_age_ = 48.45, SD_age_ = 4.34; 487 fathers, *M*_age_ = 51.22, SD_age_ = 4.92) were involved. Adolescents reported the country of birth of both parents (information that the parents themselves also reported). For the purpose of the study, only adolescents from Italian families (i.e., in which both parents were born in Italy) were included, as the focus was on youth from the majority group.

Adolescents were attending the first (48.29%) and third (51.71%) year of several high schools located in the North‐East part of Italy (i.e., Emilia‐Romagna region). Specifically, students were enrolled either in academic‐oriented (i.e., lyceum; 47.29%), technical (25.21%), vocational (12.39%), or mixed (15.10%) tracks. Most adolescents reported their parents were married or cohabiting (83.64%), while 14.49% reported their parents were separated or divorced, and the remaining (1.87%) reported other family conditions (e.g., single‐parent households). Most adolescents (79.54%) had at least one sibling, while the remaining (20.46%) were only children. Regarding parents' educational level, adolescents reported that the majority of their mothers (49.13%) and fathers (47.61%) had a medium educational level (i.e., high school diploma). Among mothers, most of the remaining (38.12%) had a high (i.e., university degree or higher), and only a few (12.75%) had a low (i.e., up to middle school diploma) educational level. Similarly, the remaining fathers had a high (27.06%) or a low (25.33%) educational level. Regarding parents' employment, most mothers (87.21%) and fathers (96.55%) reported having a job. Among mothers, the remaining were unemployed (3%) or housewives (9.62%), and only a few (0.17%) were retired. Similarly, the remaining fathers were retired (2.73%), and only a few were unemployed (0.43%) or househusbands (0.29%).

Adolescents and their parents participated in two assessments with a 1‐year interval between them (i.e., January/February 2022 and January/February 2023). All adolescents, 84.47% of their mothers, and 65.95% of the fathers participated at T1; 74.79% of adolescents, 61.11% of mothers, and 42.74% of fathers participated at T2. Little's (1988) Missing Completely at Random (MCAR) test yielded a normed *χ* ^2^ (*χ* ^2^/df = 11,349/11127) of 1.02, indicating that data were likely missing completely at random. Detailed attrition and missingness analyses are available in the Supplemental Materials section (see Tables S1a–d). Therefore, the total sample of 702 adolescents, 615 mothers, and 487 fathers was included in the analyses, and missing data were handled with the Full Information Maximum Likelihood (FIML) procedure available in M*plus* (Kelloway, 2015).

### Procedure

This longitudinal research was approved by the Ethics Committee of the Alma Mater Studiorum University of Bologna (Italy). The project involves a diverse sample of adolescents from several high schools in the North‐East part of Italy. Schools were selected through a stratified (by track and level of urbanization) randomized method, and principals were approached to present the project. Upon their approval, the study was presented to students and their parents, who received detailed oral and written information. Active consent was obtained from adolescents of age, while their underage peers provided their assent to participate in the project and active consent from their parents before participation. Participation in the study was voluntary, and students were informed they could withdraw their consent or assent at any time. At each wave, adolescents completed an online questionnaire on Qualtrics during school hours. Parents received a personalized and pseudonymized link via email to complete the questionnaire online. Adolescents and their parents were required to create a personal code (unique to each youth) to pair their answers over time and to ensure anonymity.

### Measures

#### Demographics

Participants' socio‐demographic information was collected at T1. Participants reported their age and year of school enrolment (i.e., first year and third year); sex (i.e., boys and girls); family composition (i.e., married or coliving parents, separated or divorced, other family condition and then recoded as married or coliving, divorced or other family condition); having siblings (i.e., not only child and only child); parents' educational level (i.e., no school diploma, high school diploma, bachelor's degree or master's degree and then recoded as low/medium level of education, high level of education); parents' employment (i.e., what type of work they do; then recoded as unemployed, employed).

#### Adolescents' intergroup contact at school/during leisure time

The Intergroup Contact Interactions Scale (ICIS; Karataş, Rubini, et al., 2023) validated in the Italian context was used to assess adolescents' intergroup contact. As a first question, adolescents reported the quantity of contact experienced in each context (Karataş, Rubini, et al., 2023). They answered one item (‘In the last four months, at school [e.g., with other students, teachers]/ during the activities done during your leisure time in the area you live in [e.g., your town, neighborhood], have you met and spoken with people of foreign origin?’) on a 5‐point Likert‐type rating scale ranging from 1 (*never*) to 5 (*very often*). Then, they assessed the quality of these interactions, answering 10 items scored on a 5‐point Likert‐type rating scale ranging from 1 (*never*) to 5 (*very often*). Sample items include: ‘They have been polite to you’ (positive intergroup contact; 5 items) and ‘They have been rude to you’ (negative intergroup contact; 5 items). Regarding school context, Cronbach's Alphas were 0.93 and 0.93 for positive intergroup contact and 0.83 and 0.91 for negative intergroup contact at T1 and T2, respectively. Regarding leisure time, Cronbach's Alphas were 0.93 and 0.92 for positive intergroup contact and 0.87 and 0.92 for negative intergroup contact at T1 and T2, respectively.

#### Mothers' and fathers' intergroup contact at work/during leisure time

The adapted version of the Intergroup Contact Interactions Scale (ICIS; Karataş, Rubini, et al., 2023) validated in the Italian context was used to assess parents' intergroup contact. As a first question, mothers and fathers reported the quantity of contact experienced in each context (Karataş, Rubini, et al., 2023). They answered one item (‘Over the last four months, at work [e.g., with colleagues, bosses, clients]/during the activities done during your leisure time in the area you live in [e.g., your town, neighborhood], have you met and spoken with people of foreign origin?’), on a 5‐point Likert‐type rating scale ranging from 1 (*never*) to 5 (*very often*). Then, they assessed the quality of these interactions, answering two single items (one for positive and one for negative contact; that is, ‘The experience you had with them was positive/negative?’) scored on a 5‐point Likert‐type rating scale ranging from 1 (*never*) to 5 (*very often*).

### Strategy of analyses

Descriptive, reliability, and missingness analyses were conducted using IBM SPSS Version 28.0 for Windows. The main analyses were conducted in M*plus* 8.9 (Muthén & Muthén, 2017), using the Maximum Likelihood Robust estimator (Satorra & Bentler, 2001). As a preliminary step, the longitudinal measurement invariance of adolescents' intergroup contact at school and during leisure time was tested. Scalar invariance was established for both models, as detailed in the results available in the Supplemental Materials section (see Table S3).

To test the main hypotheses of the current study (i.e., how the quality of intergroup contact is transmitted within the family context by examining the longitudinal reciprocal associations between adolescents' and their parents' positive and negative intergroup contact in different life contexts), two cross‐lagged panel models with latent variables for adolescents and observed variables for parents were tested. The first model examined cross‐lagged paths between intergroup contact quality (positive and negative) of adolescents at school and intergroup contact quality (positive and negative) of their mothers and fathers at work. The second model examined cross‐lagged paths between adolescents' intergroup contact quality (positive and negative) and their mothers' and fathers' intergroup contact quality (positive and negative) during leisure time. These models tested the longitudinal association controlling for (a) autoregressive paths (i.e., T1 → T2) and (b) within‐time correlations among all variables (i.e., correlations among variables at T1 and correlated changes at T2). In both models, the age, sex, and school type of the adolescents were added as covariates as they were expected to be associated with intergroup contact in adolescence, in line with the literature. Furthermore, preliminary and attrition analyses indicated they were indeed related to the main variables of the study.

## RESULTS

### Preliminary analyses

Means and standard deviations of study variables are reported in Table 1. Correlations among study variables are reported in Table S2 of the Supplemental Materials. Regarding the difference between structured (i.e., school and work) and unstructured (i.e., leisure time) contexts, repeated measure analyses indicated that adolescents (T1: *F =* 141.07, *p* < .001, *η* ^2^ = 0.18; T2: *F =* 99.33, *p* < .001, *η* ^2^ = 0.17), their mothers (T1: *F =* 181.44, *p* < .001, *η* ^2^ = 0.27; T2: *F =* 118.10, *p* < .001, *η* ^2^ = 0.25), and fathers (T1: *F =* 203.56, *p* < .001, *η* ^2^ = 0.32; T2: *F =* 106.11, *p* < .001, *η* ^2^ = 0.28) reported more intergroup contact in structured than in unstructured contexts. Furthermore, in the structured context, adolescents (T1: *F =* 2416.66, *p* < .001, *η* ^2^ = 0.81; T2: *F =* 876.90, *p* < .001, *η* ^2^ = 0.72), their mothers (T1: *F =* 982.99, *p* < .001, *η* ^2^ = 0.70; T2: *F =* 737.67, *p* < .001, *η* ^2^ = 0.71), and fathers (T1: *F =* 631.84, *p* < .001, *η* ^2^ = 0.63; T2: *F =* 525.21, *p* < .001, *η* ^2^ = 0.69) reported having more positive than negative contacts. The same result was found in the unstructured contexts, again for adolescents (T1: *F =* 1245.13, *p* < .001, *η* ^2^ = 0.76; T2: *F =* 397.45, *p* < .001, *η* ^2^ = 0.65), their mothers (T1: *F =* 1098.77, *p* < .001, *η* ^2^ = 0.74; T2: *F =* 836.98, *p* < .001, *η* ^2^ = 0.76), and fathers (T1: *F =* 556.73, *p* < .001, *η* ^2^ = 0.64; T2: *F =* 360.21, *p* < .001, *η* ^2^ = 0.63). Overall, these results indicate that participants had more opportunities for contact in structured (i.e., school and work) than in unstructured (i.e., leisure time) contexts, and they reported more positive than negative interactions across both contexts.

**TABLE 1:** Means (*M*) and Standard Deviations (SD) of the Study Variables.

	T1		T2	
	*M*	SD	*M*	SD
Adolescents' intergroup contact at school				
Quantity	2.78	1.25	2.53	1.33
Positive	3.98	0.73	3.94	0.71
Negative	1.52	0.61	1.78	0.80
Mothers' intergroup contact at work				
Quantity	3.22	1.23	3.15	1.24
Positive	3.94	0.66	3.94	0.67
Negative	2.03	0.81	2.00	0.81
Fathers' intergroup contact at work				
Quantity	3.18	1.23	3.20	1.13
Positive	3.83	0.73	3.86	0.07
Negative	2.03	0.84	2.08	0.79
Adolescents' intergroup contact during leisure time				
Quantity	2.15	1.16	1.90	1.15
Positive	3.91	0.70	3.80	0.70
Negative	1.64	0.71	1.94	0.85
Mothers' intergroup contact during leisure time				
Quantity	2.42	1.04	2.37	1.08
Positive	3.84	0.67	3.91	0.67
Negative	1.82	0.76	1.80	0.72
Fathers' intergroup contact during leisure time				
Quantity	2.26	1.02	2.40	1.02
Positive	3.74	0.71	3.77	0.73
Negative	1.94	0.81	1.94	0.86

### Main models

#### Cross‐lagged association between adolescents' and parents' intergroup contact in structured contexts

**FIGURE 1:**
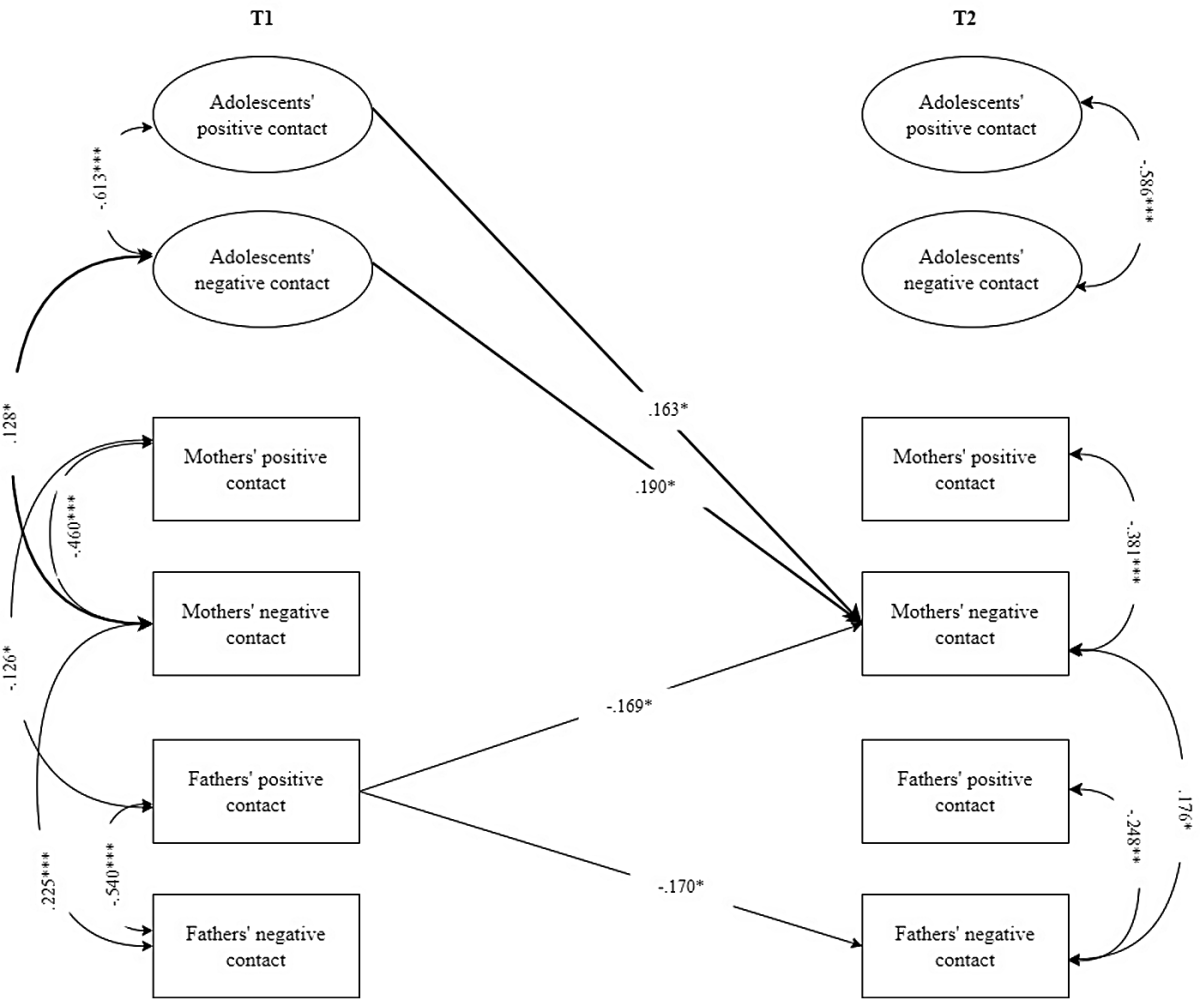
Significant standardized results of the cross‐lagged panel model of intergroup contact in structured contexts (school and work). For the sake of clarity, only significant cross‐lagged paths are displayed. Intergroup contact has been coded with higher values indicative of higher positive or negative intergroup contact. Gray arrows indicate within‐construct effects (e.g., paths between positive and negative intergroup contact of adolescents). Bold arrows indicate significant effects between adolescents' intergroup contact and parents' intergroup contact. *p < .05; **p < .01; ***p < .001.

Regarding the structured contexts (Figure 1), cross‐lagged paths indicated that adolescents' negative and positive intergroup contact at school were positively associated with mothers' negative intergroup contact at work over time. There were no significant cross‐lagged paths linking adolescents' and fathers' intergroup contact. Contrary to expectations, parents' intergroup contact quality was not significantly associated with those of adolescents at the following time point. Nevertheless, associations emerged between parents' intergroup interactions. Fathers' positive intergroup contact was negatively associated with mothers' negative intergroup contact.

Regarding within‐time correlations, adolescents' negative intergroup contact was positively correlated with mothers' negative contact at T1. Mothers' positive contact and fathers' positive contact were negatively correlated at T1. In addition, mothers' negative contact and fathers' negative contact were positively correlated both at T1 and T2.

Stability paths (see Table 2) ranged from 0.29 (*p* < .01) to 0.43 (*p* < .001) and were comparable for adolescents', mothers', and fathers' intergroup contact. Lastly, regarding covariates (see Table 2), sex was associated with adolescents' contact quality at T1 and T2 (girls reported more positive contact than boys, who reported more negative contact). Adolescents' age was associated with fathers' negative contact at T1, namely lower age of adolescents was associated with higher fathers' negative interaction. Regarding school type, attending technical/vocational schools was correlated with more negative contact for adolescents and fathers at T1, while attending academic‐oriented high schools was associated with more positive contact for fathers and mothers at T1 and for adolescents at T2.

**TABLE 2:** Standardized results of the cross‐lagged models with covariates.

Stability paths	Model 1: Intergroup contact at school and work		Model 2: Intergroup contact during leisure time	
	T1 → T2		T1 → T2	
Adolescents' positive intergroup contact	0.36***		0.42***	
Adolescents' negative intergroup contact	0.29***		0.46***	
Mothers' positive intergroup contact	0.41***		0.29**	
Mothers' negative intergroup contact	0.40***		0.37***	
Fathers' positive intergroup contact	0.40***		0.38***	
Fathers' negative intergroup contact	0.43***		0.32**	
**Covariates**	**T1**	**T2**	**T1**	**T2**
Sex → adolescents' positive intergroup contact	0.26***	0.11*	0.17***	0.11
Sex → adolescents' negative intergroup contact	−0.20***	−0.12*	−0.12*	−0.09
Sex → mothers' positive intergroup contact	−0.04	−0.04	0.03	0.06
Sex → mothers' negative intergroup contact	0.02	−0.02	−0.00	0.01
Sex → fathers' positive intergroup contact	−0.04	−0.04	0.02	0.01
Sex → fathers' negative intergroup contact	−0.01	0.07	−0.08	0.08
Age→ adolescents' positive intergroup contact	0.03	0.01	−0.20	−0.08
Age → adolescents' negative intergroup contact	−0.03	−0.01	−0.06	0.02
Age → mothers' positive intergroup contact	0.05	−0.04	−0.06	0.01
Age → mothers' negative intergroup contact	−0.05	−0.04	0.07	−0.10
Age→ fathers' positive intergroup contact	0.05	0.11	−0.02	0.04
Age → fathers' negative intergroup contact	−0.10*	0.09	0.04	0.09
School type → adolescents' positive intergroup contact	−0.05	−0.21***	−0.03	−0.05
School type → adolescents' negative intergroup contact	0.12*	0.28***	−0.03	0.10
School type → mothers' positive intergroup contact	−0.10*	−0.07	−0.04	0.04
School type → mothers' negative intergroup contact	0.05	0.02	0.00	0.00
School type → fathers' positive intergroup contact	−0.11*	−0.08	−0.11	−0.08
School type → fathers' negative intergroup contact	0.11*	−0.03	0.08	0.02

*Note*: T = Time; Sex (0 = boys, 1 = girls); Age groups (0 = first school year, 1 = third school year); School type (0 = academic‐oriented school, 1 = technical/vocational school).

Significant covariates are indicated in bold.

*p < .05, **p < .01, ***p < .001.

#### Cross‐lagged association between adolescents' and parents' intergroup contact in unstructured contexts

**FIGURE 2:**
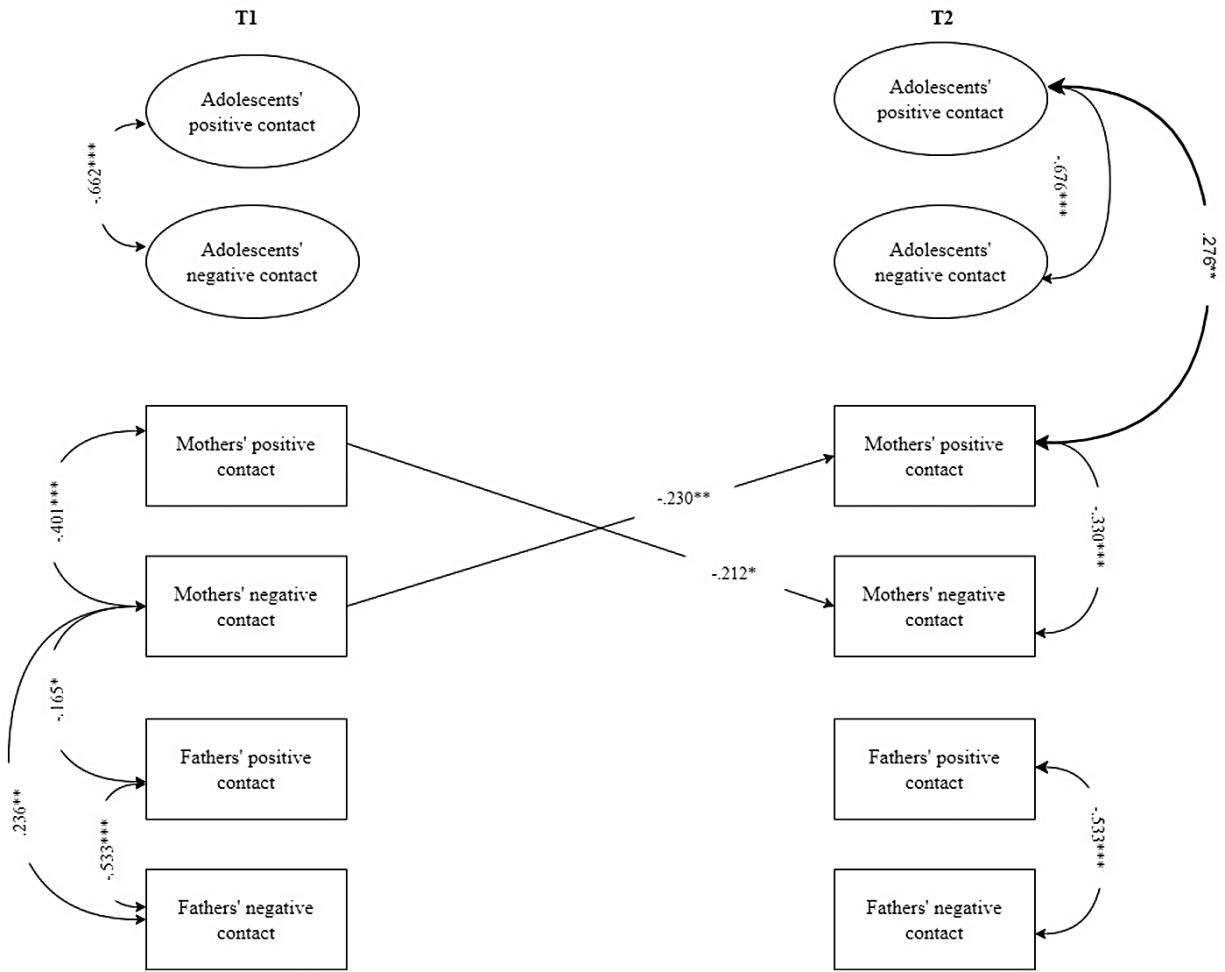
Significant standardized results of the cross‐lagged panel model of intergroup contact in unstructured contexts (leisure time). For the sake of clarity, only significant cross‐lagged paths are displayed. Intergroup contact has been coded with higher values indicative of higher positive or negative intergroup contact. Gray arrows indicate within‐construct effects (e.g., paths between positive and negative intergroup contact of adolescents). Bold arrows indicate significant effects between adolescents' intergroup contact and parents' intergroup contact. *p < .05; **p < .01; ***p < .001.

Regarding the unstructured context (i.e., leisure time), cross‐lagged paths indicated that no significant associations emerged between adolescents' intergroup contact and parents' intergroup interactions over time (Figure 2). Additionally, parents' intergroup contact quality during free time was not significantly associated with those of adolescents at the following time point.

Regarding within‐time correlations, mothers' negative intergroup contact was positively associated with fathers' negative contact at T1. Moreover, adolescents' positive intergroup contact was correlated with more positive intergroup contact of mothers at T2. Stability paths (see Table 2) ranged from 0.29 (*p* < .001) to 0.46 (*p* < .001) and were comparable for adolescents', mothers', and fathers' intergroup contact. Lastly, regarding covariates (see Table 2), sex was associated with adolescents' contact quality at T1 (i.e., girls reported more positive contact compared to boys who reported more negative contact).

## DISCUSSION

Adolescence is a crucial time in which interethnic attitudes are formed and become more stable (Wölfer et al., 2016). In addition, as today's societies are increasingly multicultural, adolescents belonging to the majority group and their families come into contact with growing ethnic and cultural diversity (Svensson & Syed, 2023). Therefore, it is important to understand how adolescents navigate such diversity, fostering positive intergroup interactions while preventing negative ones, and examine the family's role in this process. The present study advances extant knowledge on the topic by examining the role of both parents and considering different life contexts (i.e., structured and unstructured) to understand how intergroup contact in adolescence develops within the family. Overall, the results indicate that adolescents and their parents have opportunities for contact in all contexts of daily life, both in structured (where opportunities for contact are greater) and unstructured settings. Notably, adolescents' intergroup interactions at school were associated both at baseline and over time with intergroup contact with their mothers at work. In addition, changes in adolescents' and mothers' positive contacts were intertwined in the leisure contexts. From a theoretical point of view, this evidence provides a complex picture of family transmission, in which adolescents play a pivotal role in influencing their mothers. From a practical perspective, this knowledge enables the design of evidence‐based interventions involving adolescents and parents to make multicultural relationships more respectful of diversity.

### Intergenerational transmission of intergroup contact in a structured setting: The school and the work contexts

In this study, the intergroup contact quality (positive and negative) of adolescents at school and intergroup contact quality (positive and negative) of their mothers and fathers at work were first examined to understand how intergroup contact is transmitted across structured contexts. The school setting is where adolescents spend most of their time. Consequently, it is also the environment in which they are most exposed to interethnic interactions, given the increasingly multicultural composition of the classrooms (Birtel et al., 2020). In relational terms, the school can be defined as a structured context, an environment in which there are limited possibilities to choose the people with whom one enters into a relationship. The same is represented by the workplace for parents. In this view, people in structured environments are forced to interact with little choice in managing these relationships and with little opportunity to change contexts if these relationships are not fulfilling. In fact, the results of this study highlighted that adolescents and their parents report more intergroup contact in these structured settings, as compared to their experiences in their leisure time.

Furthermore, parents and adolescents reported having many positive and few negative intergroup contacts at school and work, respectively. Thus, although the people with whom they come into contact in these structured settings are contextually defined, most of the experiences are still positive. This aligns with existing literature indicating consistently that positive intergroup interactions are much more frequent than negative ones (e.g., Graf et al., 2014; Hayward et al., 2017).

Focusing on the main goal of the study, a fascinating result emerged about the association between adolescents' and parents' intergroup contact. At the beginning of the study, adolescents' negative contact and mothers' negative contact were positively correlated. Additionally, both adolescents' negative and positive intergroup contact were associated with a relative increase in mothers' negative contact over time. In contrast, no significant intergenerational associations were found regarding adolescents' and fathers' intergroup contact. These results point to two relevant considerations.

First, they indicate that contrary to expectations, no associations emerged between parents' contact at baseline and adolescent children's contact at a later time. Thus, the quality of adolescents' intergroup contact is associated over time with the quality of interactions reported by their parents, specifically by their mothers, but not the reverse. These findings highlight that effects are unidirectional, with a main child effect, rather than bidirectional. This evidence contributes to the literature on intergenerational transmission by showing that the main direction of effects depends on the nature of the aspect being transmitted. When it comes to dimensions on which parents are likely to be more stable than their adolescent children, the main direction of transmission is from parents to their adolescent children (Meeus, 2016), as documented for personality (Zentner & Renaud, 2007), self‐concept clarity (Crocetti et al., 2016), and gender role attitudes (Cano & Hofmeister, 2023). In contrast, when it comes to contextual aspects, such as the quality of intergroup contact tackled in this study, for which stability levels are generally lower than those detected for personality, attitudes, and values, and they are comparable for adolescents and their parents, the main direction of association can be from adolescents to their parents.

Second, the results showed a transmission effect concerning negative contact but not positive contact. Thus, this study confirms a more significant effect of negative valence that had already been found in studies investigating transmission in the opposite direction (from parents to adolescent children; e.g., Cernat, 2017). This is because, according to the valence‐salience effect (Paolini et al., 2010), experiencing negative contact has greater salience and makes generalization more likely than positive intergroup contact.

Finally, the results underscored that, within the parent couple, intergroup contacts of fathers and mothers were interrelated both at baseline and over time. Additionally, fathers' positive contact was associated with a relative decrease in mothers' negative contact at a later point. These results highlight that horizontally, within the couple, there is a mutual association of intergroup contact at work and, interestingly, fathers exert a more substantial influence.

### Intergenerational transmission of intergroup contact in an unstructured setting: The leisure time context

The association between intergroup contact quality (positive and negative) of adolescents and their parents during leisure time was examined to understand how intergroup contact is transmitted across unstructured contexts. While school takes up most of the adolescents' day, the rest is dedicated to leisure activities (e.g., sports and groups of friends). In relational terms, these contexts are considered unstructured because choosing people with whom one comes into contact is simpler. Adolescents can develop social and interpersonal skills in leisure contexts, characterized by an essential relational dimension (McKeown & Taylor, 2018). Thus, even in these contexts, adolescents can have different inter‐ethnic interactions. Regarding the valence of the interactions, this study showed that adolescents and their parents report more positive intergroup contact than negative. This result confirms that inter‐ethnic interactions are generally more frequently positive in both structured and unstructured contexts, in line with the literature (e.g., Graf et al., 2014; Hayward et al., 2017).

Moving into the associations between adolescents and their parents, only a positive correlated change between adolescents' and mothers' positive contact was detected. Thus, unlike the structured contexts, in the unstructured ones, there is a weaker interplay between adolescents' and their parents' intergroup contact. This result may be interpreted considering that individuals have more freedom to change relationships in unstructured contexts. Thus, they may be less affected by what happens to their family members. Overall, this evidence highlights the importance of accounting for the different developmental contexts in which adolescents are embedded (Bronfenbrenner, 1979; Sameroff, 2009).

The interplay between quality of contact within the parental couple documented for the work setting was largely confirmed also for the leisure time. This was especially the case for negative intergroup contact of mothers and fathers, which was related at baseline. These results advance the literature by showing that similarities within parental couples documented for several attitudes and values (for a review, see Luo, 2017) apply also to the quality of intergroup contact.

### Theoretical and practical implications

This study has important theoretical implications for understanding intergenerational transmission processes. Findings highlighted that, predominantly in structured settings, there is a transmission originating from adolescent children and that this process concerns only the mothers' negative contact and not the fathers. First, regarding the role of adolescents in the transmission process, this result adds to the current literature that underlines that adolescents can influence their parents, thus becoming ‘educators’ and ‘promoters’ of certain practices. This process has been called *reverse socialization*, indicating the process where parents learn knowledge, attitudes, and skills from their adolescent children (Gentina & Muratore, 2012). This happens especially for those issues for which adolescents have greater knowledge, interests (e.g., pro‐environmental problems), or skills (e.g., technology) than parents. In the domain of intergroup contact and relations, adolescents may have more to teach their parents than vice versa because they are growing up in increasingly diverse communities, unlike their parents, who may have experienced life settings with more homogeneous populations. In this regard, school plays a central role. Apart from its structural characteristics (e.g., the level of cultural diversity), it provides adolescents with formal and informal learning experiences on multiculturalism and diversity (Bayram Özdemir et al., 2024; Eckstein et al., 2021). Knowledge is a crucial factor in determining adolescents' influence on their parents. Growing up in a more multicultural society and having the opportunity to meet peers with a migrant background, especially in the school context (Karataş, Eckstein, et al., 2023b), adolescents can act as catalysts of interethnic attitudinal and behavioral changes in families. Thus, the quality of intergroup contact is a further possible object of reverse socialization, as well as pro‐environmental and technological issues.

Second, the results showed that adolescents influence the quality of intergroup contact of their mothers, but not their fathers. Mothers have always been considered crucial in the transmission process to their adolescent children (e.g., Barni et al., 2022). Additionally, the findings of the present study highlighted that mothers are also crucial in the reverse process of transmission (from their adolescent children). Adolescents generally spend more time with their mothers than with their fathers, thus having more opportunities to directly interact with them (e.g., having a discussion or sharing information; Phares et al., 2009). Additionally, they usually consider their relationship with mothers as characterized by greater support and disclosure (Alfieri et al., 2018). Responsive parents may be more prone to child influence (Knafo & Galansky, 2008) since they are willing to consider their adolescent children's points of view and readjust their practices accordingly. Similarly, Essiz and Mandrik (2022) found that intergenerational influence on sustainable consumer attitudes and behavior predominantly occurs from daughters to mothers because of the great exposure of daughters to sustainable consumer topics within their campus living environment. Unfortunately, there is a scarcity of studies considering both the mother and the father in reverse socialization processes. This could be a promising line of research development to provide a deeper understanding of family transmission mechanisms.

From a practical perspective, the results of this study allow us to consider the family transmission process during adolescence in a new light, especially for all those aspects that concern intergroup dynamics. If these findings are replicated, they can be the basis for designing evidence‐based interventions to promote positive intergroup contact. On the one hand, these interventions should involve parents along with adolescents to achieve long‐lasting attitudinal and behavioral changes in all family members. On the other hand, they should consider adolescents' active role in influencing parents' intergroup interactions. Thus, adolescents can become role models for parents on issues of inclusion and diversity (Maratia et al., 2023). This is an essential practical implication given the need to develop interventions that, especially in adolescence, can promote social cohesion and positive intergroup relationships (Reimer et al., 2021; Tropp et al., 2022).

### Limitations and suggestions for future research

The study contributed to increasing knowledge about the family transmission process of intergroup contact in adolescence, considering the role of mothers and fathers separately. Findings also highlighted the importance of considering the different life contexts in which adolescents are embedded. However, the results should be considered in light of some limitations.

First, this study allowed us to examine associations between variables over time but not to make causal inferences. Indeed, other variables may influence intergroup contact of both adolescents and their parents. For instance, living in a relatively segregated setting with few opportunities for intergroup contact could impact adolescents' intercultural experiences (Pasco et al., 2021). Thus, interpretations regarding the results should be made with caution.

Second, in this study, girls and boys reported different levels of positive and negative contact. In addition to these main differences, the interplay between adolescents' and parents' intergroup contact may unfold differently in opposite‐sex (father–daughter and mother–son) and same‐sex (father–son and mother–daughter) dyads. Although prior studies mainly documented that transmission processes were comparable in opposite‐ and same‐sex dyads (e.g., Crocetti et al., 2016 for the intergenerational transmission of self‐concept clarity), future research can delve further into the moderating role of gender groups.

Third, the present study aimed to investigate intergroup contact in adolescents belonging to the majority group. For this reason, adolescents with a migrant background were not considered. However, the transmission process may differ between families belonging to the majority or minority group, especially concerning families with a migrant background when considering intergroup dynamics such as contact (Kwak, 2003). Thus, generalizations of these results should be made with caution. To cover this gap, future studies should also examine adolescents and parents belonging to a minority group.

Finally, this longitudinal study involved two waves of data collection with a one‐year interval. So, it was not possible to capture changes in the short term. Considering that the quality of intergroup contact may be susceptible to rapid changes because it is based on daily interactions (Graf et al., 2014), future research should consider these issues and integrate the assessment of short‐, medium‐, and long‐term effects.

## CONCLUSIONS

Research has shown the importance of family in influencing the intergroup attitudes of adolescent children. However, previous studies have neglected to consider how adolescents' intergroup contact in different life contexts can be shaped within the family context. This study filled gaps in the existing literature by showing longitudinal associations between the quality of intergroup contact of adolescents and that of their mothers in different life contexts (i.e., school, work, and leisure time). Regarding the transmission process, this study highlighted that adolescents' intergroup interactions are associated with their mothers' interactions over time, not vice versa. Furthermore, considering the members of the parental couple separately highlighted the different roles of the two figures. In fact, associations emerged only with the mothers' intergroup contact. Finally, this study showed the importance of considering the valence of contact interactions and how different developmental contexts may affect intergroup contact in adolescence differently. These findings should encourage researchers to take a new look at the family transmission of intergroup contact, a look that involves adolescents as much as parents and considers intergenerational relationships also inversely (i.e., from adolescent children to parents). Operationally, this study can be the basis for future evidence‐based interventions that aim to improve intergroup relations in adolescence and make today's societies more respectful of diversity.

## FUNDING INFORMATION

This work was supported by a grant from the European Research Council (ERC) under the HORIZON EUROPE European Research Council (ERC‐CoG IDENTITIES Grant agreement N. [101002163]; Principal investigator: Elisabetta Crocetti).

## CONFLICT OF INTEREST STATEMENT

The authors report no conflict of interest.

## INFORMED CONSENT

Informed active consent was obtained from the participants' parents, and assent from the participants themselves was also collected.

## Supporting information


Table S1–S3

## Data Availability

Data that support the findings of this study are openly available in OSF at https://osf.io/8bzhw/.

## References

[jora13029-bib-0001] Alfieri, S. , Tagliabue, S. , Marta, E. , Aresi, G. , Lanz, M. , & Pozzi, M. (2018). Gratitude as a variable of mediation between parental support and self‐esteem in adolescence. Journal of Child and Family Studies, 27(5), 1394–1401. 10.1007/s10826-017-1001-4

[jora13029-bib-0002] Allport, G. W. (1954). The nature of prejudice. Addison‐Wesley.

[jora13029-bib-0003] Bagci, S. C. , & Gungor, H. (2019). Associations between perceived positive and negative parental contact and adolescent's intergroup contact experiences. International Journal of Intercultural Relations, 69, 76–86. 10.1016/j.ijintrel.2019.01.002

[jora13029-bib-0004] Bandura, A. (1977). Social learning theory. Prentice Hall.

[jora13029-bib-0005] Barni, D. , Cavazza, N. , Russo, S. , Vieno, A. , & Roccato, M. (2020). Intergroup contact and prejudice toward immigrants: A multinational, multilevel test of the moderating role of individual conservative values and cultural embeddedness. International Journal of Intercultural Relations, 75, 106–117. 10.1016/j.ijintrel.2020.02.004

[jora13029-bib-0006] Barni, D. , Rosnati, R. , & Ranieri, S. (2013). Value transmission between parents and their adolescent children: The process and its outcomes. A psycho‐social perspective. In I. Albert & D. Ferring (Eds.), Intergenerational relations. European perspectives on family and society (pp. 101–117). Policy Press.

[jora13029-bib-0007] Barni, D. , Russo, C. , Zagrean, I. , Di Fabio, M. , & Danioni, F. (2022). Adolescents' internalization of moral values: The role of paternal and maternal promotion of volitional functioning. Journal of Family Studies, 28(3), 1095–1107. 10.1080/13229400.2020.1789494

[jora13029-bib-0008] Bayram Özdemir, S. , Özdemir, M. , & Amouri, L. (2024). Unveiling the unspoken stories and experiences of pre‐service and in‐service teachers about ethnic/racial issues: Insights and directions for moving forward. *Identity*. 10.1080/15283488.2024.2404506

[jora13029-bib-0009] Birtel, M. D. , Reimer, N. K. , Wölfer, R. , & Hewstone, M. (2020). Change in school ethnic diversity and intergroup relations: The transition from segregated elementary to mixed secondary school for majority and minority students. European Journal of Social Psychology, 50(1), 160–176. 10.1002/ejsp.2609

[jora13029-bib-0010] Bronfenbrenner, U. (1979). The ecology of human development: Experiments by nature and design. Harvard University Press.

[jora13029-bib-0011] Bronfenbrenner, U. , & Morris, P. A. (2006). The bioecological model of human development. In R. M. Lerner & W. E. Damon (Eds.), Handbook of child psychology: Vol 1, Theoretical models of human development (pp. 793–828). Wiley.

[jora13029-bib-0012] Cano, T. , & Hofmeister, H. (2023). The intergenerational transmission of gender: Paternal influences on children's gender attitudes. Journal of Marriage and Family, 85(1), 193–214. 10.1111/jomf.12863

[jora13029-bib-0013] Ceccon, C. , Schachner, M. K. , Lionetti, F. , Pastore, M. , Umaña‐Taylor, A. J. , & Moscardino, U. (2023). Efficacy of a cultural adaptation of the identity project intervention among adolescents attending multiethnic classrooms in Italy: A randomized controlled trial. Child Development, 94(5), 1162–1180. 10.1111/cdev.13944 37195803

[jora13029-bib-0014] Cernat, V. (2017). Trajectories of interethnic contact and prejudice in a four‐waves study of Romanian teenager‐parent pairs. Journal of Ethnic and Migration Studies, 43(15), 2669–2687. 10.1080/1369183X.2016.1271699

[jora13029-bib-0015] Crocetti, E. , Albarello, F. , Prati, F. , & Rubini, M. (2021). Development of prejudice against immigrants and ethnic minorities in adolescence: A systematic review with meta‐analysis of longitudinal studies. Developmental Review, 60, 1–22. 10.1016/j.dr.2021.100959

[jora13029-bib-0016] Crocetti, E. , Rubini, M. , Branje, S. , Koot, H. M. , & Meeus, W. (2016). Self‐concept clarity in adolescents and parents: A six‐wave longitudinal and multi‐informant study on development and intergenerational transmission. Journal of Personality, 84(5), 580–593. 10.1111/jopy.12181 25952274

[jora13029-bib-0017] De Goede, I. H. , Branje, S. J. , & Meeus, W. H. (2009). Developmental changes in adolescents' perceptions of relationships with their parents. Journal of Youth and Adolescence, 38, 75–88. 10.1007/s10964-008-9286-7 19636793

[jora13029-bib-0018] Degner, J. , & Dalege, J. (2013). The apple does not fall far from the tree, or does it? A meta‐analysis of parent‐child similarity in intergroup attitudes. Psychological Bulletin, 139, 1270–1304. 10.1037/a0031436 23379964

[jora13029-bib-0019] Eckstein, K. , Miklikowska, M. , & Noack, P. (2021). School matters: The effects of school experiences on youth's attitudes toward immigrants. Journal of Youth and Adolescence, 50(11), 2208–2223. 10.1007/s10964-021-01497-x 34559395 PMC8505319

[jora13029-bib-0020] Essiz, O. , & Mandrik, C. (2022). Intergenerational influence on sustainable consumer attitudes and behaviors: Roles of family communication and peer influence in environmental consumer socialization. Psychology & Marketing, 39(1), 5–26. 10.1002/mar.21540

[jora13029-bib-0021] European Commission/EACEA/Eurydice . (2019). Integrating students from migrant backgrounds into schools in Europe: National policies and measures . https://eurydice.eacea.ec.europa.eu/publications/integrating‐students‐migrant‐backgrounds‐schools‐europe‐national‐policies‐and‐measures

[jora13029-bib-0022] Gentina, E. , & Muratore, I. (2012). Environmentalism at home: The process of ecological resocialization by teenagers. Journal of Consumer Behaviour, 11(2), 162–169. 10.1002/cb.373

[jora13029-bib-0023] Gniewosz, B. , & Noack, P. (2015). Parental influences on adolescents' negative attitudes toward immigrants. Journal of Youth and Adolescence, 44(9), 1787–1802. 10.1007/s10964-015-0291-3 25956291

[jora13029-bib-0024] Goodman, M. A. , & Dyer, W. J. (2020). From parent to child: Family factors that influence faith transmission. Psychology of Religion and Spirituality, 12(2), 178–190. 10.1037/rel0000283

[jora13029-bib-0025] Graf, S. , Paolini, S. , & Rubin, M. (2014). Negative intergroup contact is more influential, but positive intergroup contact is more common: Assessing contact prominence and contact prevalence in five central European countries. European Journal of Social Psychology, 44(6), 536–547. 10.1002/ejsp.2052

[jora13029-bib-0026] Hayward, L. E. , Tropp, L. E. , Hornsey, M. J. , & Barlow, K. F. (2017). Toward a comprehensive understanding of intergroup contact: Descriptions and mediators of positive and negative contact among majority and minority groups. Personality and Social Psychology Bulletin, 43(3), 347–364. 10.1177/0146167216685291 28903695

[jora13029-bib-0027] Hello, E. , Scheepers, P. , Vermulst, A. , & Gerris, J. R. M. (2004). Association between educational attainment and ethnic distance in young adults: Socialization by schools or parents? Acta Sociologica, 47(3), 253–275. 10.1177/0001699304046222

[jora13029-bib-0028] ISTAT . (2023). Stranieri residenti e nuovi cittadini: Caratteristiche demografiche e distribuzione territoriale anno 2021 . Istituto Nazionale di Statistica. https://www.istat.it/it/files//2023/03/Statistica‐Report_STRANIERI‐RESIDENTI.pdf

[jora13029-bib-0029] Jugert, P. , Eckstein, K. , Beelmann, A. , & Noack, P. (2016). Parents' influence on the development of their children's ethnic intergroup attitudes: A longitudinal analysis from middle childhood to early adolescence. European Journal of Developmental Psychology, 13(2), 213–230. 10.1080/17405629.2015.1084923

[jora13029-bib-0030] Karataş, S. , Eckstein, K. , Noack, P. , Rubini, M. , & Crocetti, E. (2023a). Positive and negative intergroup contact in school and out‐of‐school contexts: A longitudinal approach to spillover effects. Journal of Research on Adolescence, 33, 1335–1349. 10.1111/jora.12881 37688372

[jora13029-bib-0031] Karataş, S. , Eckstein, K. , Noack, P. , Rubini, M. , & Crocetti, E. (2023b). Meeting in school: Cultural diversity approaches of teachers and intergroup contact among ethnic minority and majority adolescents. Child Development, 94(1), 237–253. 10.1111/cdev.13854 36093952 PMC10086855

[jora13029-bib-0032] Karataş, S. , Rubini, M. , Prati, F. , Schwartz, S. J. , & Crocetti, E. (2023). Intergroup contact in multiple adolescents' contexts: Developing the intergroup contact interactions scale (ICIS) ‐ short version. Frontiers in Psychology, 13, 1–16. 10.3389/fpsyg.2022.1066146 PMC9875736 36710806

[jora13029-bib-0033] Kelloway, E. K. (2015). Using Mplus for structural equation modeling: A researcher's guide (2nd ed.). SAGE.

[jora13029-bib-0034] Knafo, A. , & Assor, A. (2007). Motivation for agreement with parental values: Desirable when autonomous, problematic when controlled. Motivation and Emotion, 31, 232–245. 10.1007/s11031-007-9067-8

[jora13029-bib-0035] Knafo, A. , & Galansky, N. (2008). The influence of children on their parents' values. Social and Personality Psychology Compass, 2(3), 1143–1161. 10.1111/j.17519004.2008.00097.x

[jora13029-bib-0036] Kotzur, P. F. , & Wagner, U. (2021). The dynamic relationship between contact opportunities, positive and negative intergroup contact, and prejudice: A longitudinal investigation. Journal of Personality and Social Psychology, 120(2), 418–442. 10.1037/pspi0000258 32700961

[jora13029-bib-0037] Kuczynski, L. , & Parkin, C. M. (2007). Agency and bidirectionality in socialization: Interactions, transactions, and relational dialectics. In J. E. Grusec & P. D. Hastings (Eds.), Handbook of socialization: Theory and research (pp. 259–283). Guilford Press.

[jora13029-bib-0038] Kwak, K. (2003). Adolescents and their parents: A review of intergenerational family relations for immigrant and non‐immigrant families. Human Development, 46, 115–136. 10.1159/000068581

[jora13029-bib-0039] Little, R. J. A. (1988). A test of missing completely at random for multivariate data with missing values. Journal of the American Statistical Association, 83(404), 1198–1202. 10.1080/01621459.1988.10478722

[jora13029-bib-0068] Liu, J. , Chen, Q. , & Dang, J. (2022). New intergenerational evidence on reverse socialization of environmental literacy. Sustainability Science, 17, 2543–2555. 10.1007/s11625-022-01194-z

[jora13029-bib-0040] Luo, S. (2017). Assortative mating and couple similarity: Patterns, mechanisms, and consequences. Social and Personality Psychology Compass, 11(8), e12337. 10.1111/spc3.12337

[jora13029-bib-0041] Lutterbach, S. , & Beelman, A. (2023). Relations between positive and negative extended contact experiences and prejudice in host society and refugees: Effects of positive and negative direct contact. International Journal of Intercultural Relations, 93, 1–14. 10.1016/j.ijintrel.2023.101760

[jora13029-bib-0042] Maratia, F. , Bobba, B. , & Crocetti, E. (2023). A near‐mint view toward integration: Are adolescents more inclusive than adults? Journal of Experimental Psychology: General. 10.1037/xge0001472 37747463

[jora13029-bib-0043] McKeown, S. , & Taylor, L. K. (2018). Perceived peer and school norm effects on youth antisocial and prosocial behaviours through intergroup contact in Northern Ireland. British Journal of Social Psychology, 57, 652–665. 10.1111/bjso.12257 29663432

[jora13029-bib-0044] Meeus, W. (2016). Adolescent psychosocial development: A review of longitudinal models and research. Developmental Psychology, 52(12), 1969–1993. 10.1037/dev0000243 27893243

[jora13029-bib-0045] Miklikowska, M. (2016). Like parent, like child? Development of prejudice and tolerance towards immigrants. British Journal of Psychology, 107, 95–116. 10.1111/bjop.12124 25702782

[jora13029-bib-0046] Miklikowska, M. (2017). Development of anti‐immigrant attitudes in adolescence: The role of parents, peers, intergroup friendships, and empathy. British Journal of Psychology, 108(3), 626–648. 10.1111/bjop.12236 28105654 PMC5516153

[jora13029-bib-0047] Ministero della Pubblica Istruzione . (2022). Gli alunni con cittadinanza non italiana A.S. 2020/2021 . https://www.miur.gov.it/documents/20182/0/NOTIZIARIO_Stranieri_2021+%281%29.pdf/150d451a‐45d2‐e26f‐9512‐338a98c7bb1e?t=1659103036663

[jora13029-bib-0048] Muthén, L. K. , & Muthén, B. O. (2017). Mplus user's guide (8th ed.). Muthén & Muthén.

[jora13029-bib-0049] Paolini, S. , Harwood, J. , & Rubin, M. (2010). Negative intergroup contact makes group memberships salient: Explaining why intergroup conflict endures. Personality and Social Psychology Bulletin, 36(12), 1723–1738. 10.1177/0146167210388667 21051766

[jora13029-bib-0050] Pasco, M. C. , White, R. M. B. , & Seaton, E. K. (2021). A systematic review of neighborhood ethnic–racial compositions on cultural developmental processes and experiences in adolescence. Adolescent Research Review, 6, 229–246. 10.1007/s40894-021-00152-7

[jora13029-bib-0051] Perales, F. , Hoffmann, H. , King, T. , Vidal, S. , & Baxter, J. (2021). Mothers, fathers and the intergenerational transmission of gender ideology. Social Science Research, 99, 1–14. 10.1016/j.ssresearch.2021.102597 34429210

[jora13029-bib-0052] Pettigrew, T. F. , & Tropp, L. R. (2006). A meta‐analytic test of intergroup contact theory. Journal of Personality and Social Psychology, 90(5), 751–783. 10.1037/00223514.90.5.751 16737372

[jora13029-bib-0053] Phares, V. , Fields, S. , & Kamboukos, D. (2009). Fathers' and mothers' involvement with their adolescents. Journal of Child and Family Studies, 18, 1–9. 10.1007/s10826-008-9200-7

[jora13029-bib-0054] Reimer, N. K. , Love, A. , Wölfer, R. , & Hewstone, M. (2021). Building social cohesion through intergroup contact: Evaluation of a large‐scale intervention to improve intergroup relations among adolescents. Journal of Youth and Adolescence, 50, 1049–1067. 10.1007/s10964-021-01400-8 33599936 PMC8116240

[jora13029-bib-0055] Sameroff, A. (2009). The transactional model. In A. Sameroff (Ed.), The transactional model of development: How children and contexts shape each other (pp. 3–21). American Psychological Association. 10.1037/11877-001

[jora13029-bib-0056] Satorra, A. , & Bentler, P. M. (2001). A scaled difference chi‐square test statistic for moment‐structure analysis. Psychometrika, 66(4), 507–514. 10.1007/BF02296192 PMC2905175 20640194

[jora13029-bib-0057] Schäfer, S. J. , Kauff, M. , Prati, F. , Kros, M. , Lang, T. , & Christ, O. (2021). Does negative contact undermine attempts to improve intergroup relations? Deepening the understanding of negative contact and its consequences for intergroup contact research and interventions. Journal of Social Issues, 77, 197–216. 10.1111/josi.12422

[jora13029-bib-0059] Schönpflug, U. , & Bilz, L. (2009). The transmission process: Mechanisms and contexts. In U. Schönpflug (Ed.), Cultural transmission: Psychological, developmental, social, and methodological aspects (pp. 212–239). Cambridge University Press.

[jora13029-bib-0058] Schmid, K. , Wölfer, R. , Swart, H. , Christ, O. , Al Ramiah, A. , Vertovec, S. , & Hewstone, M. (2017). The “wallpaper effect” revisited: Divergent findings on the effects of intergroup contact on attitudes in diverse versus nondiverse contexts. Personality and Social Psychology Bulletin, 43(9), 1268–1283. 10.1177/0146167217711929 28903684

[jora13029-bib-0067] Singh, P. , Sahadev, S. , Oates, C. J. , & Alevizou, P. (2020). Pro‐environmental behavior in families: A reverse socialization perspective. Journal of Business Research, 115, 110–121.

[jora13029-bib-0060] Svensson, Y. , & Syed, M. (2023). “The most exotic was the owner of the pizzeria”‐exploring the relationship between subjective diversity and ethnic identity. Identity, 23(2), 109–125. 10.1080/15283488.2022.2059663

[jora13029-bib-0061] Tropp, L. R. , White, F. , Rucinski, C. L. , & Tredoux, C. (2022). Intergroup contact and prejudice reduction: Prospects and challenges in changing youth attitudes. Review of General Psychology, 26(3), 342–360. 10.1177/10892680211046517

[jora13029-bib-0062] UNHCR . (2022). Figures at a glance . https://www.unhcr.org/figures‐at‐a‐glance.html

[jora13029-bib-0064] Wölfer, R. , Schmid, K. , Hewstone, M. , & van Zalk, M. (2016). Developmental dynamics of intergroup contact and intergroup attitudes: Long‐term effects in adolescence and early adulthood. Child Development, 87(5), 1466–1478. 10.1111/cdev.12598 27684399

[jora13029-bib-0065] Zagrean, I. , Barni, D. , Russo, C. , & Danioni, F. (2022). The family transmission of ethnic prejudice: A systematic review of research articles with adolescents. Social Sciences, 11(6), 236. 10.3390/socsci11060236

[jora13029-bib-0066] Zentner, M. , & Renaud, O. (2007). Origins of adolescents' ideal self: An intergenerational perspective. Journal of Personality and Social Psychology, 92(3), 557–574. 10.1037/0022-3514.92.3.557 17352609

