# Supplementary material for: Like parents, like children… this is not always the case! A longitudinal study on the family transmission of intergroup contact
Source: J Res Adolesc. 2024 Oct 15;34(4):1598–610. doi: 10.1111/jora.13029 (PMC11606258; doi:10.1111/jora.13029)
Supplement: Supplementary file 1 — Table S1–S3 [file JORA-34-1598-s001.docx]

**SUPPLEMENTARY INFORMATION FOR:**

Like Parents Like Children… This is not Always the Case!

A Longitudinal Study on the Family Transmission of Intergroup Contact

**Index**

**Supplementary 1:** Sample attrition analyses

**Table S1a, S1b**: Sample attrition analyses

**Table S1c, S1d**: Missingness data analyses regarding sample composition

**Supplementary 2:** Bivariate correlations

**Table S2**: Bivariate Correlations among T1 Study Variables

**Supplementary 3:** Longitudinal Measurement Invariance Tests

**Table S3:** Longitudinal Measurement Invariance Tests for Each Study Measure

**Supplementary 1: Sample attrition analyses**

A total of 702 adolescents, 615 mothers, and 487 fathers agreed to participate in the IDENTITIES project. The number of participants at T1 was as follow: 702 adolescents, 593 mothers, and 463 fathers; while at T2: 525 adolescents, 429 mothers, and 300 fathers. To gather a better understanding of the sample attrition, additional analyses were completed to ensure that the attrition was not related to specific variables by confronting adolescents who participated in the two assessments with those who attended only one assessment.

First, the effect of the demographic variables was checked for adolescents. Boys and girls were similarly represented in the two groups. In addition, the two groups are also homogeneous in terms of family composition, having siblings, parents' educational level and parents’ employment status. Conversely, a difference emerged about the type of school attended by adolescents, but no specific pattern emerged comparing observed and expected values. Additionally, adolescents who participated in both assessments were significantly younger than those who participated only in one assessment (*F*=4.713, *p*<.05, η^2^= .007) although the effect size of this difference was very small. Results are available in Table S1a and S1b.

Regarding study variables, mothers of adolescents who participated in both waves reported a lower quantity of intergroup contact at work, although the effect is very small and at the borderline of significance. No other differences emerged in relation to the study variables, thus it was possible to conclude that the different groups were rather comparable.

**Table S1a**

*Sample attrition analyses*

|  | Participated in… | | |  |  |  |  |
| --- | --- | --- | --- | --- | --- | --- | --- |
|  | 2 waves  (*n* = 525) | 1 wave  (*n* = 177) | χ^2^ | | df | *p* | Cramers’V |
| Sex |  |  | 0.124 | | 1 | .725 | .013 |
| Boys | 272  (51.81%) | 89  (50.28%) |  | |  |  |  |
| Girls | 253  (48.19%) | 88  (49.72%) |  | |  |  |  |
| School track |  |  | **7.479** | | **1** | **.006** | **.103** |
| Academic oriented | 264  (50.29%) | 68  (38.42%) |  | |  |  |  |
| Vocational/Technical | 261  (49.71%) | 109  (61.58%) |  | |  |  |  |
| Family composition |  |  | 2.268 | | 1 | .132 | .057 |
| Married or co-living | 443  (84.87%) | 140  (80%) |  | |  |  |  |
| Divorced or other | 79  (15.13%) | 35  (20%) |  | |  |  |  |
| Siblings |  |  | 0.667 | | 1 | .411 | .031 |
| Only child | 111  (21.18%) | 32  (18.29%) |  | |  |  |  |
| Not only child | 413  (78.82%) | 143  (81.71%) |  | |  |  |  |
| Mothers’ educational level |  |  | 0.345 | | 1 | .557 | .024 |
| Medium or low level | 298  (62.74%) | 91  (65.47%) |  | |  |  |  |
| High level | 177  (37.26%) | 48  (34.53%) |  | |  |  |  |
| Fathers’ educational level |  |  | 1.646 | | 1 | .200 | .058 |
| Medium or low level | 259  (69.62%) | 85  (75.89%) |  | |  |  |  |
| High level | 113  (30.38%) | 27  (24.11%) |  | |  |  |  |
| Mothers’ employment status |  |  | 0.275 | | 1 | .600 | .022 |
| Employed | 393  (88.31%) | 125  (89.93%) |  | |  |  |  |
| Unemployed | 52  (11.69%) | 14  (10.07%) |  | |  |  |  |
| Fathers’ employment status |  |  | 0.959 | | 1 | .327 | .046 |
| Employed | 334  (97.38%) | 107  (95.54%) |  | |  |  |  |
| Unemployed | 9  (2.62%) | 5  (4.46%) |  | |  |  |  |

*Notes:* Values in bold indicated that the overall effect is significant, but checking the standardized residuals the observed values of each group did not differ significantly from the expected values. Sex: 0= male, 1= female; school track: 0= academic oriented, 1= vocational/technical; family composition: 0= married or coliving, 1= divorced or other family condition; sibling: 0= not only child, 1= only child; parents’ educational level: 0= medium or low level, 1= high level; parents’ employment status: 0= unemployed; 1= employed.

**Table S1b**

*Sample attrition analyses*

|  | Participated in… | | |  |  |  |
| --- | --- | --- | --- | --- | --- | --- |
|  | 2 waves^a^  (*n* = 525) | 1 wave  (*n* = 177) | *F* | | *p* | η^2^ |
|  | *M* (SD) | *M* (SD) |  | |  |  |
| Age | 15.57 (1.11) | 15.77 (1.09) | **4.713** | | **.030** | **.007** |
| **Adolescents’ intergroup contact at school** |  |  |  | |  |  |
| Quantity | 2.68 (1.08) | 2.73 (1.32) | 0.343 | | .558 | .806 |
| Positive | 3.97 (0.64) | 3.88 (0.81) | 1.828 | | .177 | .958 |
| Negative | 1.61 (0.63) | 1.55 (0.65) | 1.171 | | .280 | .002 |
| **Mothers’ intergroup contact at work** |  |  |  | |  |  |
| Quantity | 3.15 (1.17) | 3.39 (1.17) | **4.047** | | **.045** | **.008** |
| Positive | 3.93 (0.60) | 3.99 (0.62) | 0.858 | | .355 | .002 |
| Negative | 2.00 (0.75) | 1.96 (0.77) | 0.274 | | .601 | .001 |
| **Fathers’ intergroup contact at work** |  |  |  | |  |  |
| Quantity | 3.17 (1.09) | 3.23 (1.19) | 0.222 | | .637 | .000 |
| Positive | 3.82 (0.69) | 3.89 (0.64) | 0.784 | | .376 | .002 |
| Negative | 2.06 (0.80) | 2.03 (0.72) | 0.142 | | .706 | .000 |
| **Adolescents’ intergroup contact during leisure time** |  |  |  | |  |  |
| Quantity | 2.06 (0.95) | 1.99 (1.19) | 0.597 | | .440 | .001 |
| Positive | 3.88 (0.66) | 3.80 (0.84) | 0.795 | | .373 | .002 |
| Negative | 1.74 (0.70) | 1.70 (0.80) | 0.177 | | .674 | .000 |
| **Mothers’ intergroup contact during leisure time** |  |  |  | |  |  |
| Quantity | 2.17 (0.86) | 2.08 (1.11) | 1.048 | | .306 | .002 |
| Positive | 3.84 (0.64) | 3.89 (0.60) | 0.519 | | .472 | .001 |
| Negative | 1.96 (0.74) | 1.92 (0.75) | 0.296 | | .587 | .001 |
| **Fathers’ intergroup contact during leisure time** |  |  |  | |  |  |
| Quantity | 2.32 (0.95) | 2.21 (0.98) | 0.995 | | .319 | .002 |
| Positive | 3.72 (0.68) | 3.82 (0.67) | 1.404 | | .237 | .004 |
| Negative | 1.97 (0.82) | 1.95 (0.68) | 0.054 | | .817 | .000 |

*Notes.* ^a^Means were aggregated across the two waves.

A further set of analyses were conducted to examine if there were differences according to the rate of participation of parents. Specifically, for 211 adolescents only the mother participated, for 77 only the father, and for 414 both parents participated in the study. Thus, additional analyses were completed to ensure that the sample composition was not related to specific results by confronting adolescents for whom only the mother, only the father or both parents participated.

First, the effect of the demographic variables was checked. Boys and girls were similarly represented in the three groups. In addition, the groups are also homogeneous in terms of having siblings, fathers' educational level, and parents’ employment status. Conversely, adolescents attending academic oriented schools were overrepresented in the group of adolescents with both parents participating in the study, whereas adolescents attending professional/technical school were underrepresented in the same group. Adolescents reporting that their parents are divorced were overrepresented in the group of adolescents with only the mother participating in the study and underrepresented in the group of adolescents with both parents. Additionally, a difference emerged about mothers’ educational level, but no specific pattern emerged comparing observed and expected values. Results are available in Table S1c and S1d.

Regarding study variables, adolescents with both parents participating in the study reported a lower quantity of intergroup contact at school and their fathers reported fewer positive intergroup contact at work, although the effect sizes were very small. Additionally, mothers of adolescents with both parents participating in the study, reported more negative contact at work and fewer negative contact at work, but also these effect sizes were very small. No other differences emerged in relation to the other study variables, thus it was possible to conclude that the different groups were rather comparable.

**Table S1c**

*Missingness data analyses regarding sample composition*

|  | Adolescents with… | | |  |  |  |  |
| --- | --- | --- | --- | --- | --- | --- | --- |
|  | Only mother  (*n* = 211) | Only father  (*n* = 77) | Both parents  (*n* = 414) | χ^2^ | df | *p* | Cramers’V |
| Sex |  |  |  | 0.721 | 2 | .697 | .032 |
| Boys | 106  (50.24%) | 37  (48.05%) | 218  (52.66%) |  |  |  |  |
| Girls | 105  (49.76%) | 40  (51.95%) | 196  (47.34%) |  |  |  |  |
| School type |  |  |  | **21.072** | **2** | **<.001** | **.173** |
| Lyceum | 82  (38.86%) | 25  (32.47%) | **225 (+)**  **(54.35%)** |  |  |  |  |
| Professional/Technical | 109  (61.14%) | 52  (67.53%) | **189 (-)**  **(45.65%)** |  |  |  |  |
| Family composition |  |  |  | **21.562** | **2** | **<.001** | **.176** |
| Married or co-living | 159  (75.36%) | 58  (77.33%) | 366  (89.05%) |  |  |  |  |
| Divorced or other | **52 (+)**  **(24.64%)** | 17  (22.67%) | **45 (-)**  **(10.95%)** |  |  |  |  |
| Siblings |  |  |  | 2.131 | 2 | .344 | .055 |
| Only child | 40  (18.96%) | 12  (15.58%) | 91  (22.14%) |  |  |  |  |
| Not only child | 171  (81.04) | 65  (84.42%) | 320 (77.86%) |  |  |  |  |
| Mothers’ educational level |  |  |  | **7.849** | **1** | **.005** | **.113** |
| Medium or low level | 145 (71.08%) |  | 244 (59.51%) |  |  |  |  |
| High level | 59  (28.92%) |  | 166 (40.49%) |  |  |  |  |
| Fathers’ educational level |  |  |  | 0.220 | 1 | .639 | .021 |
| Medium or low level |  | 55  (73.33%) | 289 (70.66%) |  |  |  |  |
| High level |  | 20  (26.67%) | 120 (29.34%) |  |  |  |  |
| Mothers’ employment status |  |  |  | 0.184 | 2 | .912 | .018 |
| Employed | 22  (12.02%) |  | 338  (88.95) |  |  |  |  |
| Unemployed | 161  (87.98%) |  | 42  (11.05%) |  |  |  |  |
| Fathers’ employment status |  |  |  | 2.673 | 2 | .263 | .077 |
| Employed |  | 63  (100%) | 341 (96.60%) |  |  |  |  |
| Unemployed |  | 0 | 12 (3.40%) |  |  |  |  |

*Notes:* Values in bold indicated that overall effect is significant, but neither group differs significantly from expected values. When observed values are significantly different from expected values, (+) indicates that the observed value is higher than the expected value, while (-) indicates that the observed value is lower than the expected value. Sex: 0= male, 1= female; school track: 0= academic oriented, 1= vocational/technical; family composition: 0= married or coliving, 1= divorced or other family condition; sibling: 0= not only child, 1= only child; parents’ educational level: 0= medium or low level, 1= high level; parents’ employment status: 0= unemployed; 1= employed.

**Table S1d**

*Missingness data analyses regarding sample composition*

|  | Adolescents with… | | |  |  |  |
| --- | --- | --- | --- | --- | --- | --- |
|  | Only mother  (*n* = 211) | Only father  (*n* = 77) | Both parents  (*n* = 414) | *F* | *p* | η^2^ |
|  | *M* (SD) | *M* (SD) | *M* (SD) |  |  |  |
| Age | 15.65 (1.15) | 15.74 (1.10) | 15.58 (1.09) | .803 | .448 | .002 |
| **Adolescents’ intergroup contact at school** |  |  |  |  |  |  |
| Quantity | 2.82 (1.20) | 2.88 (1.24) | 2.59 (1.09) | **4.105** | **.017** | **.012** |
| Positive | 3.99 (0.67) | 3.96 (0.59) | 3.93 (0.70) | 0.507 | .603 | .002 |
| Negative | 1.61 (0.68) | 1.61 (.06) | 1.59 (0.62) | 0.094 | .910 | .000 |
| **Mothers’ intergroup contact at work** |  |  |  |  |  |  |
| Quantity | 3.34 (1.20) |  | 3.14 (1.16) | 3.492 | .062 | .007 |
| Positive | 3.98 (0.59) |  | 3.93 (0.62) | 0.857 | .355 | .002 |
| Negative | 1.83 (0.75) |  | 2.07 (0.74) | **10.583** | **.001** | **.022** |
| **Fathers’ intergroup contact at work** |  |  |  |  |  |  |
| Quantity |  | 3.08 (1.29) | 3.20 (1.07) | 0.660 | .417 | .001 |
| Positive |  | 3.99 (0.66) | 3.01 (0.68) | **3.884** | **.049** | **.009** |
| Negative |  | 1.93 (0.76) | 2.05 (0.78) | 1.928 | .166 | .005 |
| **Adolescents’ intergroup contact during leisure time** |  |  |  |  |  |  |
| Quantity | 2.14 (1.08) | 1.99 (1.05) | 1.99 (0.96) | 1.470 | .231 | .004 |
| Positive | 3.87 (0.75) | 3.88 (0.80) | 3.87 (0.65) | 0.011 | .989 | .000 |
| Negative | 1.71 (0.76) | 1.83 (0.78) | 1.72 (0.69) | 0.570 | .566 | .003 |
| **Mothers’ intergroup contact during leisure time** |  |  |  |  |  |  |
| Quantity | 2.24 (1.00) |  | 1.99 (1.05) | 2.257 | .105 | .007 |
| Positive | 3.89 (0.71) |  | 3.83 (0.58) | 0.702 | .403 | .002 |
| Negative | 1.83 (0.76) |  | 2.01 (0.73) | **6.008** | **.015** | **.012** |
| **Fathers’ intergroup contact during leisure time** |  |  |  |  |  |  |
| Quantity |  | 2.41 (1.13) | 2.27 (0.92) | 1.253 | .264 | .003 |
| Positive |  | 3.83 (0.76) | 3.73 (0.66) | 1.117 | .291 | .003 |
| Negative |  | 1.99 (0.70) | 1.96 (0.80) | 0.60 | .807 | .000 |

*Notes.* Means were aggregated across the four waves. Means with different subscripts differ significantly (*p* < .05).

**Supplementary 2: Bivariate Correlations**

**Table S2**

*Bivariate Correlations among T1 Study Variables*

|  | 1. | 2. | 3. | 4. | 5. | 6. | 7. | 8. | 9. | 10. | 11. | 12. | 13. | 14. | 15. | 16. | 17. | 18. |
| --- | --- | --- | --- | --- | --- | --- | --- | --- | --- | --- | --- | --- | --- | --- | --- | --- | --- | --- |
| 1. Adolescents’ contact quantity at school |  |  |  |  |  |  |  |  |  |  |  |  |  |  |  |  |  |  |
| 2. Adolescents’ positive contact at school | .16^***^ |  |  |  |  |  |  |  |  |  |  |  |  |  |  |  |  |  |
| 3. Adolescents’ negative contact at school | .02 | -.58^***^ |  |  |  |  |  |  |  |  |  |  |  |  |  |  |  |  |
| 4. Mothers’ contact quantity at work | .11^*^ | -.12^*^ | .10 |  |  |  |  |  |  |  |  |  |  |  |  |  |  |  |
| 5. Mothers’ positive contact at work | .07 | .08 | -.08 | .12^*^ |  |  |  |  |  |  |  |  |  |  |  |  |  |  |
| 6. Mothers’ negative contact at work | -.05 | -.03 | .13^*^ | .14^**^ | -.46^***^ |  |  |  |  |  |  |  |  |  |  |  |  |  |
| 7. Fathers’ contact quantity at work | .03 | .02 | -.05 | .04 | -.07 | .04 |  |  |  |  |  |  |  |  |  |  |  |  |
| 8. Fathers’ positive contact at work | -.04 | .04 | -.01 | -.02 | .04 | -.11 | .18^**^ |  |  |  |  |  |  |  |  |  |  |  |
| 9. Fathers’ negative contact at work | .05 | -.03 | .06 | .10 | -.10 | .24^***^ | .09 | -.54^***^ |  |  |  |  |  |  |  |  |  |  |
| 10. Adolescents’ contact quantity during leisure time | .33^***^ | .10^*^ | .07 | .08 | -.01 | .01 | -.02 | -.06 | .03 |  |  |  |  |  |  |  |  |  |
| 11. Adolescents’ positive contact during leisure time | .17^***^ | .54^***^ | -.45^***^ | -.04 | .06 | -.19^**^ | .02 | .14^*^ | -.10 | .23^***^ |  |  |  |  |  |  |  |  |
| 12. Adolescents’ negative contact during leisure time | -.10 | -.44^***^ | .56^***^ | .02 | -.10 | .17^*^ | -.02 | -.18^**^ | .23^***^ | -.06 | -.56^***^ |  |  |  |  |  |  |  |
| 13. Mothers’ contact quantity during leisure time | .08 | -.02 | -.04 | .35^***^ | .17^**^ | -.05 | .05 | .07 | -.01 | .13^**^ | .10 | -.02 |  |  |  |  |  |  |
| 14. Mothers’ positive contact during leisure time | .02 | .06 | -.12^*^ | .01 | .42^**^ | -.26^***^ | -.02 | .09 | -.04 | -.04 | -.06 | .03 | -.03 |  |  |  |  |  |
| 15. Mothers’ negative contact during leisure time | .02 | -.11 | .09 | .01 | -.36^***^ | .59^**^ | .04 | -.01 | .21^**^ | -.05 | -.13 | .10 | -.04 | -.40^***^ |  |  |  |  |
| 16. Fathers’ contact quantity during leisure time | .03 | .05 | -.07 | .04 | .01 | -.01 | .32^**^ | .16^**^ | -.09 | .10^*^ | .06 | -.01 | .10^*^ | .02 | -.03 |  |  |  |
| 17. Fathers’ positive contact during leisure time | -.07 | .05 | -.11 | .03 | .02 | -.18* | .04 | .54^***^ | -.33^***^ | -.02 | .07 | -.06 | -.05 | .13 | -.18^*^ | .29^***^ |  |  |
| 18. Fathers’ negative contact during leisure time | .05 | -.14^*^ | .16^*^ | .05 | -.14 | .20^**^ | -.02 | -.45^***^ | .59^***^ | .04 | -.14 | .12 | .06 | -.13 | .26^***^ | -.05 | -.57^***^ |  |

*Note.* ^*^ *p* < .05, ^**^ *p* < .01, ^***^ *p* < .001

**Longitudinal Measurement Invariance Tests**

**Table S3**

*Longitudinal Measurement Invariance Tests for Each Study Measure*

|  | **Model fit indices** | | | | | **Model comparison** | | | | | |
| --- | --- | --- | --- | --- | --- | --- | --- | --- | --- | --- | --- |
|  | *χ*_SB_^2^ | *df* | CFI | SRMR | RMSEA [90% CI] | Models | Δχ_SB_^2^ | Δ*df* | *p* | ΔCFI | ΔRMSEA |
| **Intergroup contact at school** | | | | | |  |  |  |  |  |  |
| M1. Configural model | 305.041 | 154 | .962 | .043 | .040 [.034, .047] |  |  |  |  |  |  |
| M2. Metric model | 315.314 | 162 | .961 | .048 | .039 [.033, .046] | M2-M1 | 10.465 | 8 | .234 | -.001 | -.001 |
| M3. Full scalar model | 368.895 | 172 | .961 | .062 | .043 [.037, .050] | M3-M2 | 70.196 | 10 | .000 | -.001 | .004 |
| M3a. Partial scalar model^1^ | 358.867 | 171 | .953 | .062 | .043 [.036, .049] | M3-M2 | 55.295 | 9 | .000 | -.008 | .004 |
| **Intergroup contact during leisure time** | | | | | |  |  |  |  |  |  |
| M1. Configural model | 256.713 | 154 | .965 | .046 | .038 [.030, .046] |  |  |  |  |  |  |
| M2. Metric model | 269.819 | 162 | .963 | .053 | .038 [.030, .046] | M2-M1 | 13.114 | 8 | .108 | -.002 | .000 |
| M3. Full scalar model | 309.862 | 172 | .953 | .069 | .042 [.034, .049] | M3-M2 | 48.485 | 10 | .000 | -.010 | .004 |
| M3a. Partial scalar model^1^ | 307.893 | 171 | .954 | .068 | .042 [.034, .049] | M3-M2 | 46.364 | 9 | .000 | -.009 | .004 |

*Note.* χ_SB_^2^ = Satorra-Bentler scaled chi-square; *df* = degrees of freedom; CFI = Comparative Fit Index; TLI = Tucker-Lewis Index; SRMR = Standardized Root Mean Square Residual; RMSEA [90% CI] = Root Mean Square Error of Approximation and 90% Confidence Interval; Δ = Change in the parameter.

^1^ Partial scalar invariance was achieved for the intergroup contact scale both at school and during leisure time: intercept 2 for the first model and intercept 8 for the second model were not constrained to be equal across waves.
